# Social Determinants of Health and What Mothers Say They Need and Want After Release From Jail

**DOI:** 10.5888/pcd15.180260

**Published:** 2018-12-06

**Authors:** Elisabeth Stelson, Marjie Mogul, Holly Harner, Jeane Ann Grisso, Rosemary Frasso

**Affiliations:** 1 Department of Social and Behavioral Sciences, T.H. Chan School of Public Health, Harvard University, Boston, Massachusetts; 2 Department of Research and Evaluation, Maternity Care Coalition, Philadelphia, Pennsylvania; 3 Public Health Program, School of Nursing and Health Sciences, LaSalle University, Philadelphia, Pennsylvania; 4 Center for Public Health Initiatives, University of Pennsylvania, Philadelphia, Pennsylvania; 5 College of Population Health, Thomas Jefferson University, Philadelphia, Pennsylvania

## Abstract

Identifying the biopsychosocial needs of mothers who have been released from jail is critical to understanding the best ways to support their health and stability after release. In May through August 2014, we interviewed 15 mothers who had been released from an urban jail about their reentry experiences, and we analyzed transcripts for themes. Eight domains of community reentry emerged through analysis: behavioral health services, education, employment, housing, material resources, medical care, relationships with children, and social support. Participants defined barriers to successful reentry, which paralleled the social determinants of health, and shared suggestions that could be used to mitigate these barriers.

## Objective

From 1970 to 2014, the number of incarcerated women in local jails rose 14-fold, from 8,000 to 110,000 (1). Nearly all research on incarceration, including qualitative research, focuses on state and federal prisons (1–3). Jails — operated by municipalities to hold pre-sentenced and nonfelony inmates for shorter stays — are largely overlooked (1,4). Fifty-two percent of jailed women have at least 1 physical health problem, 32% have serious mental illness, 82% have substance-use disorders, and 63% to 80% are mothers of minor children (1,5). Most jailed women are released back into their communities (6). Identifying factors in the community that influence their social determinants of health is critical because these factors correlate with individual health outcomes (7,8).

## Methods

In May through August 2014, we recruited from a maternal–child case management (MCCM) program a convenience sample of mothers who had been released within the previous 25 months from a large, urban jail in the mid-Atlantic. Participants were aged 18 years or older, spoke English, had been released for a least 1 month, and had at least 1 child younger than 4 years at the time of release (an MCCM program participation requirement). Child custody was not required. Pregnant women and women receiving court-stipulated behavioral health treatment were ineligible to participate.

This qualitative study consisted of one-time, semistructured interviews. Case managers from the MCCM program, members of the MCCM parent organization’s community advisory board, and external incarceration experts reviewed the interview guide and provided feedback. The interview guide focused on mothers’ community-reentry experience, relationships with their children, future goals, and suggestions to improve the reentry experience. Participants chose the interview location. Interviews lasted from 60 to 150 minutes and were audiorecorded. This study was approved by the University of Pennsylvania Institutional Review Board.

Interviews were transcribed verbatim, checked for accuracy, de-identified, and entered into NVivo version 10 (QSR International Pty Ltd.), a software program that facilitates qualitative analysis. The research team used a directed content approach for analysis and generated a codebook consisting of several a priori codes and other codes generated from the open coding process (9). We used the constant comparative method to review the consistency of internal transcript coding and coding between transcripts (10). The first author (E.S.) coded all 15 transcripts, 6 of the 15 were double-coded by 2 members of the study team (M.M. and J.A.G.), and the senior author (R.F.) audited the coded transcripts. Intercoder reliability averaged 95%. We grouped codes into domains, which closely matched the constructs of social determinants of health identified by the World Health Organization (7). We extracted and tabulated at least 1 illustrative comment for each domain when participants provided an idea for improving the reentry experience. 

## Results

Of the 137 mothers identified, 95 (69%) could not be reached, 26 (19%) did not meet eligibility requirements, and 1 declined participation. Fifteen participants completed interviews; the average age was 28 (Table 1).

**Table 1:** Characteristics of Participants (N = 15) in a Qualitative Study on the Reentry Experiences of Mothers Released From an Urban Jail, 2014^a^

Characteristic	Mean (Range)
Age, y	28 (20–37)
Age of participants’ children	7.2 y (1 mo–20 y)
No. of children	3 (1–7)
Duration of incarceration	5.5 mo (21 d–1.5 y)
Time since release, mo	8.8 (1.5–25.0)

a Of the 15 participants, 10 were African American, 3 were non-Hispanic white, and 2 were Hispanic.

Eight domains of community reentry emerged through transcript analysis: behavioral health services for mental health and substance use, education, employment, housing, material resources, medical care, relationships with children, and social support (Table 2). Participants frequently mentioned visiting their primary care provider or their child’s pediatrician within the first few weeks of release, noting few barriers to care. In contrast, participants provided numerous examples of barriers to stable and healthy reentry that corresponded with the other 7 domains. For example, housing was a barrier. Participants explained how strained familial relationships made it difficult to share housing, but participants could not afford housing on their own because of difficulty securing employment. This barrier was compounded by limited access to supportive housing programs. Many programs were full, and participants were afraid for their safety and of losing custody of their children if they accessed emergency shelter.

**Table 2:** Domains of Community Reentry, Barriers, and Participant-Generated Solutions, Qualitative Study on the Reentry Experiences of Mothers Released From an Urban Jail, 2014

Domain of Community Reentry	Barriers to Reentry	Example of Participant-Generated Solutions
Behavioral health services	• Long waitlist for treatment • No insurance if participant did not have custody of children • Inadequate treatment at in-patient facilities • Difficulty meeting the program attendance requirements due to competing demands	**Professional support to manage reentry stress:** “Just to talk to somebody. Like I said, this was my first time getting locked up so I wasn’t used to coming home. I wasn’t prepared for it really. I just had to deal with it the way I thought would be best. So I guess having somebody to talk to about it would have been better.”
Education	• Competing time demands of work, childcare, and behavioral health treatment • Welfare provides childcare subsidies only if full-time student or part-time student and working, but there are other time demands during reentry • Financial expenses of school and perceived ineligibility for federal student loans with a misdemeanor or felony • Limited access to internet and quiet study space	**Financial support for GED and higher education:** “Single moms can’t afford [GED courses]! It’s hard enough affording stuff for the baby. So it’s like if you get your GED and you actually pass it, we’ll waive all the fees. Once you pass it, these are the schools that will accept you, and financial aid and everything — that would help so much.”
Employment	• Discrimination against persons with a criminal record • Personal discouragement in job search • Limited community resources to help with job search • Mental health problems • Competing demands (eg, caring for children and school)	**Require probation officers to help link clients to jobs:** “My probation officer . . . she doesn’t have any resources to help me find a job, even though that was a stipulation from my sentence — find a job. . . . She should be there to help me stay out of jail. Ain’t that the point — helping me staying out of jail by finding a job?”
Housing	• Unstable familial and romantic relationships jeopardize cohabitation • Difficulty affording rent due to limited economic opportunity with a criminal record • Public supportive housing programs are at capacity • Discomfort with the emergency shelter system (ie, perceived to be unsafe, having too many rules, and/or resembles incarceration)	**Transitional housing to bridge reentry process: **“If they would come up with a reentry program type of house just for girls coming out that don’t have any help. . . . For a lot of girls, when you get released from jail and you’re on the streets and you have nothing, of course you are going to go back to drugs or prostitution or whatever it is they do.”
Material resources	• Some agencies and nonprofit organizations have strict eligibility requirements to receive donations (eg, diapers, clothes, household goods). • Some agencies do not provide strong follow-up with services • Fear that reporting material hardship to agencies would be equated with neglecting their children	**Increased access to donations:** “I’d say some type of service where it would make it . . . someone coming out of jail with no money, being able to get freebies, handouts. . . . There was nothing when I came out. I didn’t have clothes.”
Medical care	• No barriers identified by participants, and access to care was frequently mentioned (eg, “The first thing I did when I came home was I went to the doctors and got a checkup. I went to see my doctor.”)	None provided by participants
Relationship with children	• Coordination with Child Protective Services • Lack of stable housing • Unstable relationships with children’s caregiver • Time elapsed during incarceration	**House arrest instead of incarceration:** “You had your baby in jail. You missed out on the first couple of years just for them to find you innocent [because you cannot afford bail]. You know, they could have let you be at home. Put you on house arrest — put an ankle bracelet on them, something. . . . I think they should be able to be outside and be in the world and have their children and get to be there for their children.”
Social support	• Minimal social support from peers • Inconsistent social support from family members and partners	**Mentorship and peer support opportunities: **“I think maybe if I had like a mentor or somebody to help me . . . Maybe when I came home, if I had somebody that was like, ‘Okay, so what is your goals?’ . . . If I had somebody that stays on top of me and keeps in touch with me more.”

Suggestions for improving reentry mostly related to the 8 domains identified. For example, a participant suggested that inconsistent social support from family and friends (voiced by many participants) could be remedied by a formalized peer support program: “I think maybe if I had a mentor or somebody to help me. . . . Maybe when I came home, if I had somebody that was like, ‘Okay, so what are your goals?’ . . . If I had somebody that stays on top of me and keeps in touch with me more.”

## Discussion

Study participants identified numerous social and economic barriers to reentry that can affect health and stability (7), a greater number than identified in most prison studies (2,3). When organized into domains, these barriers reflected the proximal and intermediate social determinants of health recognized by the World Health Organization. Participants rarely spoke of distal social determinants of health, such as political or economic forces in their communities (7). With the exception of access to medical care, for which participants noted few obstacles, the domains were often interrelated, exacerbating the effect of individual barriers. For example, mothers found it difficult to regain custody of children without stable housing, which was challenging to secure without steady income and employment. Throughout the interviews, participants shared suggestions for mitigating these barriers.

This study has several limitations. Women released from jail are a transient population, so our sample size was small and did not reach saturation. Because participants were recruited from an MCCM program, selection bias may have influenced responses to questions on access to medical care and social support. Stigma surrounding incarceration may have also introduced social desirability bias. Nevertheless, when reached, mothers rarely declined to participate; they were racially representative of the jailed population of the city (11) and described consistent experiences across the sample.

A qualitative approach to learning what mothers value and need is essential to ensuring that health and social services match these values and needs (12). As social determinants of health, the barriers identified in this study potentially affect immediate postrelease stability and the long-term health and well-being of mothers and children (6). Because most jailed women are mothers of minor children, investment in supportive reentry services that address the domains in this study has the potential to improve the health of children and their previously incarcerated mothers.
